# Supplementary material for: A phase 2a clinical trial of molnupiravir in patients with COVID-19 shows accelerated SARS-CoV-2 RNA clearance and elimination of infectious virus
Source: Sci Transl Med. 2022 Jan 19;14(628):eabl7430. doi: 10.1126/scitranslmed.abl7430 (PMC10763622; doi:10.1126/scitranslmed.abl7430)
Supplement: 20211223-1 [file scitranslmed.abl7430.v1.pdf]

Cite as: W. A. Fischer *et al.*, *Sci. Transl. Med.* 10.1126/scitranslmed.abl7430 (2021).

CORONAVIRUS

A Phase 2a clinical trial of Molnupiravir in patients with COVID-19 shows accelerated SARS-CoV-2 RNA clearance and elimination of infectious virus

William A. Fischer II[1]*, Joseph J. Eron Jr[2], Wayne Holman[3], Myron S. Cohen[1], Lei Fang[4], Laura J. Szewczyk[3], Timothy P. Sheahan[5], Ralph Baric[5], Katie R. Mollan[6], Cameron R. Wolfe[7], Elizabeth R. Duke[8], Masoud M. Azizad[9], Katyna Borroto-Esoda[10], David A. Wohl[1], Robert W. Coombs[12], Amy James Loftis[1], Paul Alabanza[11], Felicia Lipansky[3], and Wendy P. Painter[3]*

[1]Institute for Global Health and Infectious Disease, Division of Pulmonary Diseases and Critical Care Medicine, The University of North Carolina at Chapel Hill; Chapel Hill, NC, USA. [2]Department of Medicine, Division of Infectious Diseases, The University of North Carolina at Chapel Hill; Chapel Hill, NC, USA. [3]Ridgeback Biotherapeutics LP; Miami, FL, USA. [4]Pharstat Inc.; Raleigh, NC, USA. [5]Department of Epidemiology, The University of North Carolina at Chapel Hill; Chapel Hill, NC, USA. [6]Gillings School of Global Public Health, School of Medicine, The University of North Carolina at Chapel Hill; Chapel Hill, NC, USA. [7]Department of Medicine, Division of Infectious Diseases, Duke University Medical Center; Durham, NC, USA. [8]Fred Hutchinson Cancer Research Center, University of Washington; Seattle, WA, USA. [9]Valley Clinical Trials, Inc.; Northridge, CA, USA. [10]KBE Consulting; Raleigh, NC, USA. [11]Lineberger Comprehensive Cancer Center, The University of North Carolina at Chapel Hill; Chapel Hill, NC, USA. [12]Department Laboratory Medicine & Pathology, University of Washington; Seattle, WA, USA

*Corresponding author. Email: William_Fischer@med.unc.edu and Painter@Ridgebackbio.com

There is an urgent need for an effective, oral, direct-acting therapeutic to block transmission of severe acute respiratory syndrome coronavirus 2 (SARS-CoV-2) and to prevent progression to severe coronavirus disease-2019 (COVID-19). In a Phase 2a double-blind, placebo-controlled, randomized, multicenter clinical trial, we evaluated the safety, tolerability, and antiviral efficacy of the nucleoside analog molnupiravir in 202 unvaccinated participants with confirmed SARS-CoV-2 infection and with symptom duration <7 days. Participants were randomized 1:1 to receive 200 mg molnupiravir or placebo, and then 3:1 to receive molnupiravir (400 or 800 mg) or placebo, orally twice daily for 5 days. Antiviral activity was assessed by reverse transcriptase-polymerase chain reaction (RT-PCR) for SARS-CoV-2 RNA in nasopharyngeal swabs. Infectious virus was assessed by inoculation of cultured Vero cells with samples from nasopharyngeal swabs and was detected by RT-PCR. Time to viral RNA clearance (primary endpoint) was decreased in the 800 mg molnupiravir group (median 14 days) compared to the placebo group (median 15 days) (log rank p-value=0.013). 92.5% of participants receiving 800 mg molnupiravir achieved viral RNA clearance compared with 80.3% of placebo recipients by study end (4 weeks). Infectious virus (secondary endpoint) was detected in swabs from 1.9% of the 800 mg molnupiravir group compared with 16.7% of placebo group at day 3 of treatment (p =0.016). At day 5 of treatment, infectious virus was not isolated from any participants receiving 400 or 800 mg molnupiravir compared with 11.1% of placebo recipients (p =0.034 and 0.027, respectively). Molnupiravir was well tolerated, with a similar number of adverse events across all doses.

INTRODUCTION

Severe acute respiratory syndrome coronavirus 2 (SARS-CoV-2), the virus responsible for coronavirus disease-2019 (COVID-19), has caused more than 268,934,575 confirmed infections and 5,297,850 deaths worldwide as of 12 December 2021 (*1*). Studies have shown associations between high SARS-CoV-2 nasopharyngeal RNA and both the isolation of infectious (replication-competent) virus and hospitalization rates (*2*–*7*). Additionally, animal studies have reported similar associations between viral RNA load and transmission (*8*). Currently, two classes of antiviral drugs are authorized for use in the treatment of COVID-19 including remdesivir, a nucleoside analog, and three different monoclonal antibody products directed against the SARS-CoV-2 Spike protein including bamlanivimab and etesivimab, casirivimab and imdevimab, and sotrovimab. Remdesivir is approved by the US Food and Drug Administration (FDA) for the treatment of hospitalized patients with severe COVID-19. The monoclonal antibodies are allowed under emergency use authorization (EUA) for treating high-risk outpatients with mild-to-moderate COVID-19 within 10 days of symptom onset. The monoclonal antibodies were granted EUA based on data from randomized controlled trials demonstrating a reduction in viral RNA replication and a decreased risk of progression of illness, but logistical challenges have limited access to these treatments (*2*, *9*, *10*). Furthermore, no therapies have yet been

shown to hasten the elimination of infectious virus and prevent transmission. Thus, there is an urgent need for oral antiviral drug therapies that can be easily distributed on a scale that meets global demand, and that reduce disease progression and prevent SARS-CoV-2 transmission.

Molnupiravir, the prodrug of the ribonucleoside analog β-D-N^4-hydroxycytidine (NHC), is rapidly converted in plasma to NHC and then to the active 5′-triphosphate form by host cell kinases (*11*, *12*). The active 5′-triphosphate serves as a competitive substrate for virally-encoded RNA-dependent RNA polymerase (RdRp), and once incorporated into nascent viral RNA, induces an antiviral effect via accumulation of errors that increase with each viral replication cycle (*13*–*15*). Preclinical studies revealed broad-spectrum antiviral activity against coronaviruses, including SARS-CoV-2, with a high barrier to resistance (*16*). In humanized mouse models, molnupiravir treatment and prophylaxis reduced the replication and pathogenesis of SARS-CoV, SARS-CoV-2, high-risk SARS-like bat coronaviruses, and Middle East respiratory syndrome coronavirus (MERS-CoV) (*17*). In a ferret model of SARS-CoV-2 infection, molnupiravir treatment completely blocked transmission of virus to untreated animals, suggesting that early treatment could potentially prevent secondary spread of SARS-CoV-2 (*8*).

Molnupiravir has been shown to be safe and well tolerated in a first-in-human Phase 1 trial in healthy volunteers (*13*). Herein, we report the results of a Phase 2a, double-blind, placebo-controlled, randomized, multicenter clinical trial designed to evaluate the safety, tolerability, and antiviral activity of molnupiravir dosed twice daily (BID) for 5 days in patients with mild to moderate COVID-19. Hypotheses included that molnupiravir would be safe, well tolerated and decrease the time to clearance of SARS-CoV-2 RNA (primary endpoints), as well as reduce infectious SARS-CoV-2 in nasopharyngeal swabs (secondary endpoint) in adults with symptomatic SARS-CoV-2 infection.

RESULTS

Participant Demographics and Clinical Characteristics

Two hundred and four participants were randomized with 202 receiving at least 1 dose of molnupiravir or placebo between 19 June 2020 and 21 January 2021 at 10 sites in the United States (Fig. 1; Table 1). Seven participants discontinued participation due to adverse events or physician-decision/participant withdrawal or were lost to follow-up. Overall, the participants randomized to each dose of molnupiravir and placebo were well matched, however, the baseline prevalence of antibodies to SARS-CoV-2 Spike protein was greater in the 800 mg molnupiravir group (35.3%) compared with the placebo group (18.2%; Table 1).

Time to Clearance of SARS-CoV-2 Viral RNA in Nasopharyngeal Swabs

Time to clearance of viral RNA in nasopharyngeal swabs (<1018 copies/mL) was the primary endpoint of this study and was significantly reduced in participants receiving 800 mg molnupiravir (median 14 days) compared with those administered placebo (median 15 days) (log-rank p-value = 0.013; Table 2 and Fig. 2A). Time to clearance of viral RNA was not significantly different in participants who received 200 mg or 400 mg molnupiravir compared to those who received placebo (Fig. 2A). Given the difference in baseline antibody status between those who received molnupiravir 800 mg and placebo, sensitivity analysis evaluating only those participants who were seronegative at baseline was performed. The reduction in time to clearance of viral RNA between 800 mg molnupiravir and placebo in the seronegative participants was greater and remained significant (median 14 days versus 27 days, respectively; p = 0.001) (Table S1). The proportion of participants who achieved SARS-CoV-2 RNA clearance by day 28 (end of study) was also greater for those administered 800 mg molnupiravir (92.5%) compared with those administered 200 mg molnupiravir (91.3%), 400 mg molnupiravir (78.7%), or placebo (80.3%) (Table 2).

Safety and Tolerability of Molnupiravir

Molnupiravir was associated with few, and mainly low-grade, adverse events (Table 3 and Table S2) that were similar to those reported by participants assigned to the placebo group. The incidence of treatment-associated adverse events was lowest in the 800 mg molnupiravir group. The only adverse events reported by more than 4 participants were headache, insomnia, and increased alanine aminotransferase and there was no difference by treatment arm or dose. Two (1.4%) adverse events led to discontinuation of molnupiravir compared with one adverse event (1.6%) for placebo. Grade 3 or higher adverse events occurred in 5.0% and 8.1% of the combined molnupiravir groups and the placebo group, respectively. There were no dose-related trends in hematology or clinical chemistry data during the study.

Four serious adverse events occurred and resulted in hospitalization: one participant (1.6%) in the placebo group had hypoxia, two participants (3.2%) in the 400 mg molnupiravir group including one with a cerebrovascular accident and one with decreased oxygen saturation, and one participant (1.8%) in the 800 mg molnupiravir group had acute respiratory failure (Table 3). Treatment was discontinued early in 3 of the 4 participants. The participant in the placebo group who experienced the serious adverse event of hypoxia died 31 days after onset of the serious adverse event.

Isolation of Infectious SARS-CoV-2 Virus from Nasopharyngeal Swabs

The secondary virological endpoint of this study was isolation of infectious virus from nasopharyngeal swabs and the reduction in time (days) to negativity. Infectious virus was isolated from 43.5% (74/170) of evaluable nasopharyngeal

swabs at baseline (Table 2 and Fig. 2B). On day 3 of treatment, infectious virus was isolated from only one of 53 (1.9%) participants administered 800 mg molnupiravir compared with 9 of 54 (16.7%) participants administered placebo (p = 0.016). Infectious virus isolation also decreased on day 5 of treatment in participants administered 400 or 800 mg molnupiravir, with no infectious virus isolated from participants in these groups (0/42 and 0/53, respectively) compared with 11.1% (6/54) of placebo recipients (p = 0.034 and 0.027, respectively) (Fig. 2B). The difference in infectious virus isolation remained significant for the 400 and 800 mg molnupiravir groups (compared with placebo, p =0.03 and 0.02, respectively) on day 5 of treatment, when analysis was limited to those participants with infectious virus isolated at baseline (Table S3, p = 0.03 and 0.03, respectively). The difference in infectious virus isolation also remained significant for the 400 and 800 mg molnupiravir groups (compared with placebo) on day 5 when the analysis was limited to those participants who were seronegative for SARS-CoV-2 and who had infectious virus present in nasopharyngeal swabs at baseline (Table S4). Additionally, the difference in the proportion of participants with infectious virus isolated on day 5 remained significant for the 800 mg dose of molnupiravir when compared with concurrently enrolled placebo recipients (Table S5, p = 0.014). Overall, there was a significant dose-response relationship in infectious virus isolation, as determined by the exact Cochran-Armitage trend test, with the proportion of participants with infectious virus isolated lower in the 400 or 800 mg molnupiravir groups compared with the 200 mg molnupiravir group or the placebo group on day 3 (p = 0.010) and day 5 (p = 0.003) (Table 2). The dose response was also significant on day 5 when analysis was limited to participants who had infectious virus isolated at baseline (p = 0.004; Table S3).

Change in SARS-CoV-2 Viral Load from Baseline

The decrease in viral RNA from baseline at study days 3, 5, 7, and 14 was greater for the 800 mg molnupiravir group than for any of the other groups at each time point (Table 2 and Fig. 2C). For participants administered 400 or 800 mg molnupiravir, the least squares mean viral load change from baseline was significantly greater on day 5 when compared with the placebo group, with differences in least squares means of -0.434 and -0.547 $\log_{10}$ copies/mL (p = 0.030 and 0.006), respectively. Additionally, for participants administered 800 mg molnupiravir, the least squares mean viral load change from baseline was also significantly greater on day 7 compared with the placebo group, with a least squares mean difference of -0.534 $\log_{10}$ copies/mL (p = 0.006). The reduction in viral load from baseline to day 5 between the 800 mg molnupiravir and placebo groups remained significant during sensitivity analyses limited to participants who were negative for antibodies to SARS-CoV-2 Spike protein at baseline (least squares mean difference of -0.613 $\log_{10}$ copies/mL; p = 0.002; Table S6) and when compared to concurrently enrolled participants given placebo (least squares mean difference of -0.376 $\log_{10}$ copies/mL; p = 0.045; Table S7).

Clinical Outcomes

This study did not preferentially enroll high risk individuals, and was not powered to detect a difference in hospitalization. However, daily symptom diaries were collected to evaluate severity of symptoms, time to resolution of symptoms, and overall health associated with COVID-19. At baseline, a greater proportion of participants administered molnupiravir reported their health as poor or fair (68.3%) compared with those who received placebo (50.0%). The median time to resolution of COVID-19 symptoms was not statistically different between participants who received molnupiravir and those who received placebo: 9.0 days (95% confidence interval [CI] 6.0, 13.0), 5.5 days (95% CI 4.0, 8.0), 8 days (95% CI 6.0, 12.0), and 8.5 days (95% CI 7.0, 11.0) in the 200 mg, 400 mg, 800 mg molnupiravir groups and placebo recipients, respectively. The median time to participant-reported general health of at least "very good" was similar between the pooled molnupiravir groups (14 days; 95% CI 13, 21) and placebo group (15 days; 95% CI 12.0, 18.0) (Table S8). Overall, 12 (8.6%) participants receiving molnupiravir and 5 (8.1%) participants receiving placebo required a COVID-19 associated medical visit (Table S9), which was not a statistically significant difference.

SARS-CoV-2 Antibody Detection

Participants were tested for SARS-CoV-2 Spike protein-specific immunoglobulin IgA, IgM, and IgG at baseline and on days 7 and 28 of the study. The proportions of participants with any antibody to SARS-CoV-2 Spike protein at baseline varied between the groups, with 15.0%, 30.0%, 35.3%, and 18.2% in the 200 mg, 400 mg, and 800 mg molnupiravir and placebo groups, respectively. By day 28, 99.2% of molnupiravir-treated participants had developed antibodies to SARS-CoV-2 Spike protein, compared with 96.5% of those administered placebo.

Next Generation Sequencing of SARS-CoV-2 RNA-Dependent RNA Polymerase

Genotypic changes in the RdRp occurred at a higher rate among participants who received molnupiravir compared with placebo. On average, 5.7 nucleotide changes in the RdRp were observed following treatment with placebo. By comparison, the average number of nucleotide changes in RdRp following molnupiravir treatment was two-fold greater (10.9), which was statistically significant (p = 0.024). This supported viral error induction as the mechanism of action of molnupiravir (*15*).

DISCUSSION

In this Phase 2a randomized, double-blind, placebo-controlled clinical trial, molnupiravir was well tolerated and was

associated with antiviral efficacy. This was shown by reduced infectious virus isolation from nasopharyngeal swabs, reduced time to elimination of SARS-CoV-2 RNA, an increased proportion of participants who cleared SARS-CoV-2 RNA, and a greater reduction in SARS-CoV-2 viral RNA from baseline compared with those given placebo, in a cohort of outpatients with COVID-19. Four days after treatment initiation, there was no infectious virus isolated from any participants who received 400 or 800 mg of molnupiravir. The findings from this study support a recent interim analysis of data from a Phase 3 clinical trial of molnupiravir in adult outpatients with mild-to-moderate COVID-19. The interim analysis reported a significant decrease in rates of hospitalization and death from 14.1% among placebo recipients to 7.3% among participants treated with 800 mg molnupiravir. The combined analysis, including all randomized participants, revealed a continued decrease in hospitalization and death among those treated with molnupiravir (6.8%) compared with those who received placebo (9.7%). Importantly, there was also an 89% (95% CI, 14-99) reduction in the risk of death among participants who received molnupiravir compared with placebo, suggesting that early control of viral replication prevented progression to severe illness and death (*18*).

In our phase 2a clinical trial, the decrease in infectious virus isolation, reduction in the time to clearance of viral RNA, the greater proportion of participants who eliminated SARS-CoV-2 viral RNA, and the greater change in viral RNA from baseline in participants treated with 800 mg molnupiravir compared with placebo support the antiviral efficacy of molnupiravir at the 800 mg dose. These results remained significant even when accounting for differences in time from symptom onset, viral RNA, and seropositivity. Importantly, only 26.1% (46/176) of all participants had evidence of a humoral immune response to SARS-CoV-2 at baseline, indicating that clinical trial randomization of participants occurred early in the clinical course of infection. However, by day 28, 99.2% (131/132) of those who received any dose of molnupiravir were antibody positive, demonstrating that early treatment with molnupiravir had an antiviral effect without inhibiting the development of an anti-SARS-CoV-2 Spike protein antibody response.

The primary endpoint of this Phase 2a study was the median time to viral clearance, and the difference between participants who received 800 mg molnupiravir compared with placebo was one day. Importantly, in the Kaplan-Meier plots of time to clearance of SARS-CoV-2 viral RNA, there was continued separation between the 800 mg molnupiravir group and the placebo group resulting in a greater proportion of individuals who received 800 mg molnupiravir clearing SARS-CoV-2 viral RNA by the end of the study (day 28) compared with placebo recipients (92.5% vs 80.3%, $p = 0.013$). Additionally, when the analysis was limited to seronegative participants, who were likely earlier in their course of infection compared with seropositive participants, the difference in the median time to viral clearance between the 800 mg and placebo groups was even greater: 14 days versus 27 days, respectively ($p = 0.001$). Taken together, these results demonstrate that treatment with 800 mg molnupiravir was associated with an accelerated clearance of viral RNA.

In clinical trials of COVID-19 therapeutics, antiviral efficacy against SARS-CoV-2 has been assessed by measuring changes in viral RNA, however, detection of viral RNA does not confirm the replication competence of the virus. We believe demonstration of the ability of treatment to reduce and eliminate infectious SARS-CoV-2 is an essential consideration. In observational studies, SARS-CoV-2 virus isolation decreases with time, and persistence of infectious virus is associated with disease severity and host immune status (*4*, *19*, *20*). In otherwise healthy adults with mild to moderate COVID-19, infectious virus has been isolated up to 10 days after symptom onset (*3*, *7*, *19*, *20*). In our phase 2a trial, infectious SARS-CoV-2 was isolated from 11.1% of placebo recipients at day 5 of the study, with a mean of 9 to 10 days from symptom onset. Treatment with molnupiravir accelerated the clearance of infectious virus, with no infectious virus isolated on day 5 from participants who received 400 mg or 800 mg molnupiravir. In this study, the evaluation of antiviral activity extended beyond clearance of viral RNA and demonstrated clearance of infectious virus in participants with COVID-19 treated with molnupiravir, which has important clinical implications for progression of illness and virus transmission. These findings may also have implications for the use of molnupiravir after exposure to SARS-CoV-2, which is being investigated in an ongoing Phase 3 randomized double-blind, placebo-controlled study to evaluate the efficacy and safety of molnupiravir for post-exposure prophylaxis in adults residing with a person with COVID-19 (ClinicalTrials.gov Identifier: NCT04939428).

Safety analyses from this Phase 2a trial were consistent with those from a Phase 1 trial of molnupiravir and support ongoing clinical development (*13*). Overall, molnupiravir was well tolerated with no increase in treatment-related or serious adverse events compared with participants administered placebo. There were no safety signals or evidence of hematologic, renal, or hepatic toxicity at any dose.

As a nucleoside analog, molnupiravir acts by increasing the incorporation of errors into viral RNA beyond an acceptable limit leading to elimination of viral replication. Given this mechanism of action and the ability of this compound to induce nucleotide changes in the Ames assay, whole animal mutagenicity assays were conducted and mutation rates were not found to be different between molnupiravir-treated animals and untreated control animals (*21*).

Importantly, this Phase 2a clinical trial was designed to

evaluate the antiviral efficacy and safety of molnupiravir and was not powered to evaluate clinical endpoints, such as symptom duration or hospitalization, nor was enrollment limited to participants with risk factors for severe COVID-19. However, a Phase 2/3 study is ongoing to evaluate the effect of molnupiravir on symptom duration and severity, emergency department visits, and hospitalizations (ClinicalTrials.gov Identifier: NCT04575597) (*18*).

An important limitation of our phase 2a trial was the imbalance in the randomization, with a greater proportion of seropositive individuals and a lower viral load at baseline among those randomized to receive 800 mg molnupiravir compared to placebo. To determine if these baseline differences affected the differences in viral RNA elimination, infectious virus clearance and change in viral load from baseline, sensitivity analyses were conducted using participants who were seronegative at baseline or who had infectious virus isolated at baseline. Differences in infectious virus isolation at day 5, time to clearance of viral RNA, and reductions in viral load from baseline to day 5 remained significant when analysis was limited to participants who were antibody negative at baseline. Among participants who were negative for antibodies at baseline, there was no difference in baseline viral load between the 800 mg molnupiravir group and the placebo group.

An additional limitation was the use of a pooled placebo group in the analyses. In this clinical trial, participants were enrolled from 19 Jun 2020 through 21 Jan 2021. According to the U.S. Centers for Disease Control, SARS-CoV-2 variants of concern began to emerge in early 2021, at the very end of enrollment of this study (*22*). The alpha variant (B.1.1.7), which was first reported in the United Kingdom in December 2020, was first reported in the United States on 9 Jan 2021 but did not become a predominant strain until after enrollment was completed. Importantly, differences in virus isolation and viral load reduction on day 5 of the study remained significant when analysis was limited to comparisons between 800 mg molnupiravir and concurrently enrolled placebo participants (Table S1). These sensitivity analyses indicated that the antiviral efficacy of molnupiravir demonstrated in this study was not due to imbalances in seropositivity, trends in viral RNA at baseline, or the use of a pooled placebo group. Additionally, this study was largely conducted prior to emergency use authorization of COVID-19 vaccines, which limited the evaluation of antiviral activity in individuals with breakthrough infections. Given the mechanism of action of molnupiravir, it is unlikely that antiviral efficacy would be decreased in the setting of active viral replication in a vaccinated individual.

Three combination intravenous monoclonal antibody products have been granted emergency use authorization by the U.S. FDA in ambulatory patients with COVID-19 to reduce medical visits and hospitalizations. However, the logistical challenges of intravenous and subcutaneous therapies limit timely administration to patients who may benefit most from these important treatments (*2*, *9*, *10*, *23*). Unfortunately, the perpetual evolution of SARS-CoV-2 variants of concern has resulted in origination of viruses that can evade neutralization by some monoclonal antibodies (*24*). Unlike Spike protein, which varies widely across the coronavirus family and has accumulated mutations throughout the pandemic, the viral target for molnupiravir's antiviral activity is the highly conserved viral RdRp. RdRp may be less plastic and does not have the same capacity for evolutionary change as Spike protein does. Moreover, molnupiravir has broad antiviral activity against many genetically unrelated coronaviruses including recent SARS-CoV-2 variants of concern (*25*, *26*). Importantly, studies thus far have shown that coronaviruses have a high barrier to resistance to the parental nucleoside of molnupiravir (*15*). Collectively, these data suggest that the emergence of future SARS-CoV-2 variants of concern is unlikely to diminish the antiviral activity of molnupiravir as the target of its antiviral activity has a diminished capacity for change compared to Spike protein.

This phase 2a trial provides strong biological evidence supporting development of molnupiravir as an oral agent to reduce infectious virus replication and interrupt progression of COVID-19 during early stages of disease. Current evidence suggests that uninterrupted viral replication is a major sign of progression to more severe disease (*2*, *5*). Critically, molnupiravir can be produced at scale and does not require cold transportation or infection control infrastructure for administration. The results of this trial demonstrate the safety, tolerability, and antiviral efficacy of molnupiravir to reduce replication of SARS-CoV-2 and accelerate clearance of infectious virus and support ongoing clinical trials of molnupiravir.

MATERIALS AND METHODS

Study Design

We conducted a Phase 2a randomized, double-blind, placebo-controlled trial of multiple doses of molnupiravir in non-hospitalized adults with recently diagnosed COVID-19 (ClinicalTrials.gov Identifier: NCT04405570). Unvaccinated adults aged ≥18 years were eligible if they had a positive test for SARS-CoV-2 infection within 96 hours and had onset of symptoms of COVID-19 within 7 days at the time of treatment initiation (day 1). Antiviral activity, safety, and tolerability of molnupiravir were assessed for 28 days following treatment initiation. Nasopharyngeal swabs were collected on days 1 (baseline), 3, 5, 7, 14, and 28 for measurement of antiviral activity by detection of SARS-CoV-2 RNA using RT-PCR and by isolation of infectious virus from nasopharyngeal swabs. Participants were followed for 28 days to assess safety, with assessments performed on days 1, 3, 5, 7, 14, and 28; adverse events were monitored throughout the study period. The

primary safety and tolerability objectives assessed adverse events that were Grade 3 or higher and those that led to early treatment discontinuation. Severity and duration of self-reported COVID-19 symptoms (a secondary objective) were collected daily using a symptom diary. Plasma samples were collected to evaluate immune response and serology using a qualitative antigen-capture enzyme-linked immunosorbent assay (*28–30*).

This trial complied with the Declaration of Helsinki, the International Council on Harmonisation Guidelines for Good Clinical Practice, and applicable local regulations. The protocol was approved by a central Institutional Review Board (Western IRB/WCG IRB, Tracking No: 20201509) and written informed consent was obtained from each of the 240 participants prior to study entry.

Randomization and Intervention

This study was initiated as a single center, single dose study which was subsequently amended to allow dose escalation as clinical development of molnupiravir proceeded. Participants were initially randomized 1:1 to 200 mg molnupiravir or matching placebo, n = 46. As the safety experience from the Phase 1 study became available, this Phase 2 study was amended to expand to multiple study sites and to a dose definition design with additional cohorts added at 400 mg or 800 mg randomized at 3:1. Doses were administered orally BID for 5 days. Initiation of the next higher dose group occurred following review of safety and virology data from this and other studies of molnupiravir. Molnupiravir (100 mg and 200 mg) and matching placebo were supplied as dry filled capsules for oral administration. Doses administered in this study were 200 mg, 400 mg, or 800 mg BID.

Participants were randomized using the REDCap randomization application (The University of North Carolina at Chapel Hill Translational and Clinical Sciences Institute and REDCap Development Team). The randomization schedule was generated using PROC PLAN in SAS Version 9.4 (SAS Institute Inc., Cary, NC) by an independent statistician. Randomization was at a 1:1 ratio (molnupiravir:placebo) in Part 1 and was stratified by time since onset of COVID-19 symptoms (early [0 to ≤60 hours] versus late [>60 to ≤168 hours]). Randomization was at a 3:1 ratio (molnupiravir:placebo) in Parts 2 to 9 and was not stratified. The randomization in Part 1 used 6 blocks of size 2 plus 8 blocks of size 4 to generate a total of 44 allocations to each of the early and late strata, in case either of the strata enrolled all of the participants in Part 1. An additional 44 randomization allocations were appended to the end of each stratum (for a total of 88 in each stratum) using the same permuted block design to allow for approved replacement or over-enrollment. The randomization in Parts 2 to 9 used a fixed block size of 4 to generate either 4 blocks of size 4, 5 blocks of size 4, or 6 blocks of size 4 (ie, 16, 20, or 24 participants), depending on the requested number of assignments. An additional 4 randomization allocations were appended to each part (for a total of 20 across Parts 2 to 9) for approved replacement or over-enrollment. Within- and between-block seeds were randomly generated in SAS Version 9.4 prior to initialization of the random number generator. Participants with missing infectivity and SARS-CoV-2 RNA data were imputed based on the algorithms provided in Table S10 and Table S11. The investigators, subjects, and sponsor were blinded to the treatments received. However, a subset of sponsor representatives was unblinded to the treatment randomization to facilitate ongoing review of virology data.

Antiviral Efficacy

The primary antiviral efficacy outcome was time to viral RNA clearance, as measured by quantitative RT-PCR analysis of nasopharyngeal swabs using a validated, laboratory-developed test based on the U.S. Centers for Disease Control and Prevention 2019-nCoV Emergency Use Authorization assay. Time to viral RNA clearance was defined as the first of two timepoints where viral RNA was below the limit of quantitation (<1,018 copies/mL). If the first negative test occurred on the last on-study assessment, it was considered to have achieved viral RNA clearance on the last assessment. Participants who completed the study without achieving viral RNA clearance were censored at the last viral RNA assessment. Participants who discontinued the study without achieving viral RNA clearance were censored at day 28.

Secondary antiviral efficacy outcomes were time to infectious virus elimination from nasopharyngeal swabs and median viral RNA change from baseline on days 3, 5, 7, 14, and 28. Infectious virus isolation was performed using Vero C1008 cells (Vero 76, clone E6, Vero E6 from ATCC) and was assessed by quantitative RT-PCR of viral RNA in culture medium at 2 and 5 days post inoculation (*3*, *27*). Samples were considered positive if the supernatant had at least 1,000 copies/mL SARS-CoV-2 RNA at 2 days post inoculation or if the relative fold-change in RNA copy number from culture days 2 to 5 post inoculation was >5. The criteria for positivity were created based on preliminary studies and recombinant virus controls of 50 PFU and 500 PFU included for every batch of clinical samples. The 5-fold increase metric was selected a priori to control for the possibility of RNA contamination from input samples or RNA from defective virus that did not increase over time as it was replication defective. No detectable RNA contamination from input samples was detected.

The “greater than 1000 copies” metric was selected a priori to account for positive samples that had very high viral RNA on day 2 post-inoculation in this assay (i.e., ~10^7 copies/mL), consistent with saturation and meeting or exceeding the upper limit of detection, which would not allow for an increase greater than 5-fold by day 5 post-inoculation in the assay. All samples positive for infectious virus by this assay

had intra-assay copy numbers of RNA well above the 1000 copy threshold. The limit of quantitation in the RT-PCR assay to detect infectious virus was 200 copies/mL. Three nasopharyngeal swabs (2 from 400 mg molnupiravir-treated participants who tested negative and 1 from a placebo participant who tested positive) were analyzed despite having been received out of temperature range.

Viral Genome Sequencing

Next generation sequencing of SARS-CoV-2 polymerase RdRp was performed by Monogram Biosciences on samples collected at baseline and on day 5 to analyze nucleotide changes and confirm the mechanism of action.

Statistical Analyses

Time to response for viral RNA negativity was summarized using Kaplan-Meier methodology. Median time to response and cumulative probability of response by visit (with 95% CI) were analyzed by treatment group. Comparisons of treatment effects were performed using log-rank tests. The number and percentage of subjects who were negative for infectious virus isolation were summarized and between-group comparisons were conducted using Fisher's exact test. Dose-response assessments were performed using the exact Cochran-Armitage trend test.

The primary endpoint was evaluated a priori using a Kaplan-Meier estimation with a corresponding exact log-rank test. Anticipating up to 10% missing data, 44 participants (approximately 22 for active and placebo) provided 88% power to detect a risk difference (active – placebo) of -35% in the primary endpoint using a type I error rate (alpha) of 0.10. The sample size in the dose escalation part was based on assessment of the primary endpoint across all parts. A total of 96 active and 32 placebo participants would achieve 83% power to detect a between-group difference of ≥0.6 standard deviations, with a type I error rate (alpha) of 0.05 using a two-sided test.

Treatment comparisons between active drug and placebo groups for SARS-CoV-2 nasopharyngeal viral load change from baseline were analyzed using a mixed model for repeated measures, with restricted maximum likelihood estimation and an unstructured covariance matrix. The model included fixed effects of treatment, study visit, days since COVID-19 symptom onset, and baseline SARS-CoV-2 viral load ($\log_{10}$ copies/mL); and interaction terms of treatment by visit, days since COVID-19 symptom onset by visit, and baseline SARS-CoV-2 viral load by visit. The estimated mean treatment difference for active minus placebo at each visit is presented with the 95% CIs and corresponding p values.

Comparisons of next-generation sequencing data between treatments were performed using a two-sample t test, based on the average number of treatment-emergent nucleotide changes. Analyses were conducted using SAS Version 9.4 (SAS Institute Inc., Cary NC) and two-sided tests were performed using an alpha of 0.05 for treatment comparisons. Adjustments for multiple testing were not performed.

SUPPLEMENTARY MATERIALS

www.science.org/doi/10.1126/scitranslmed.abl7430
Tables S1 to S11
MDAR Reproducibility Checklist

REFERENCES AND NOTES

1. World Health Organisation, Weekly epidemiological update on COVID-19. Edition 70, published 14 December 2021.– [Internet]. Coronavirus Dis. COVID-19 Pandemic. [cited 2021 DEC 14]; Available from: https://www.who.int/publications/m/item/weekly-epidemiological-update-on-covid-19---14-december-2021
2. P. Chen, A. Nirula, B. Heller, R. L. Gottlieb, J. Boscia, J. Morris, G. Huhn, J. Cardona, B. Mocherla, V. Stosor, I. Shawa, A. C. Adams, J. Van Naarden, K. L. Custer, L. Shen, M. Durante, G. Oakley, A. E. Schade, J. Sabo, D. R. Patel, P. Klekotka, D. M. Skovronsky; BLAZE-1 Investigators, SARS-CoV-2 Neutralizing Antibody LY-CoV555 in Outpatients with Covid-19. *N. Engl. J. Med.* **384**, 229–237 (2021). doi:10.1056/NEJMoa2029849 Medline
3. R. Wölfel, V. M. Corman, W. Guggemos, M. Seilmaier, S. Zange, M. A. Müller, D. Niemeyer, T. C. Jones, P. Vollmar, C. Rothe, M. Hoelscher, T. Bleicker, S. Brünink, J. Schneider, R. Ehmann, K. Zwirglmaier, C. Drosten, C. Wendtner, Author Correction: Virological assessment of hospitalized patients with COVID-2019. *Nature* **588**, E35–E35 (2020). doi:10.1038/s41586-020-2984-3 Medline
4. J. J. A. van Kampen, D. A. M. C. van de Vijver, P. L. A. Fraaij, B. L. Haagmans, M. M. Lamers, N. Okba, J. P. C. van den Akker, H. Endeman, D. A. M. P. J. Gommers, J. J. Cornelissen, R. A. S. Hoek, M. M. van der Eerden, D. A. Hesselink, H. J. Metselaar, A. Verbon, J. E. M. de Steenwinkel, G. I. Aron, E. C. M. van Gorp, S. van Boheemen, J. C. Voermans, C. A. B. Boucher, R. Molenkamp, M. P. G. Koopmans, C. Geurtsvankessel, A. A. van der Eijk, Duration and key determinants of infectious virus shedding in hospitalized patients with coronavirus disease-2019 (COVID-19). *Nat. Commun.* **12**, 267 (2021). doi:10.1038/s41467-020-20568-4 Medline
5. Y. Liu, L.-M. Yan, L. Wan, T.-X. Xiang, A. Le, J.-M. Liu, M. Peiris, L. L. M. Poon, W. Zhang, Viral dynamics in mild and severe cases of COVID-19. *Lancet Infect. Dis.* **20**, 656–657 (2020). doi:10.1016/S1473-3099(20)30232-2 Medline
6. J. Chen, T. Qi, L. Liu, Y. Ling, Z. Qian, T. Li, F. Li, Q. Xu, Y. Zhang, S. Xu, Z. Song, Y. Zeng, Y. Shen, Y. Shi, T. Zhu, H. Lu, Clinical progression of patients with COVID-19 in Shanghai, China. *J. Infect.* **80**, e1–e6 (2020). doi:10.1016/j.jinf.2020.03.004 Medline
7. J. Bullard, K. Dust, D. Funk, J. E. Strong, D. Alexander, L. Garnett, C. Boodman, A. Bello, A. Hedley, Z. Schiffman, K. Doan, N. Bastien, Y. Li, P. G. Van Caeseele, G. Poliquin, Predicting Infectious Severe Acute Respiratory Syndrome Coronavirus 2 From Diagnostic Samples. *Clin. Infect. Dis.* **71**, 2663–2666 (2020). doi:10.1093/cid/ciaa638 Medline
8. R. M. Cox, J. D. Wolf, R. K. Plemper, Therapeutically administered ribonucleoside analogue MK-4482/EIDD-2801 blocks SARS-CoV-2 transmission in ferrets. *Nat. Microbiol.* **6**, 11–18 (2021). doi:10.1038/s41564-020-00835-2 Medline
9. R. L. Gottlieb, A. Nirula, P. Chen, J. Boscia, B. Heller, J. Morris, G. Huhn, J. Cardona, B. Mocherla, V. Stosor, I. Shawa, P. Kumar, A. C. Adams, J. Van Naarden, K. L. Custer, M. Durante, G. Oakley, A. E. Schade, T. R. Holzer, P. J. Ebert, R. E. Higgs, N. L. Kallewaard, J. Sabo, D. R. Patel, P. Klekotka, L. Shen, D. M. Skovronsky, Effect of Bamlanivimab as Monotherapy or in Combination With Etesevimab on Viral Load in Patients With Mild to Moderate COVID-19: A Randomized Clinical Trial. *JAMA* **325**, 632–644 (2021). doi:10.1001/jama.2021.0202 Medline
10. D. M. Weinreich, S. Sivapalasingam, T. Norton, S. Ali, H. Gao, R. Bhore, B. J. Musser, Y. Soo, D. Rofail, J. Im, C. Perry, C. Pan, R. Hosain, A. Mahmood, J. D. Davis, K. C. Turner, A. T. Hooper, J. D. Hamilton, A. Baum, C. A. Kyratsous, Y. Kim, A. Cook, W. Kampman, A. Kohli, Y. Sachdeva, X. Graber, B. Kowal, T. DiCioccio, N. Stahl, L. Lipsich, N. Braunstein, G. Herman, G. D. Yancopoulos; Trial Investigators, REGN-COV2, a Neutralizing Antibody Cocktail, in Outpatients with Covid-19. *N. Engl. J. Med.* **384**, 238–251 (2021). doi:10.1056/NEJMoa2035002 Medline
11. F. Kabinger, C. Stiller, J. Schmitzová, C. Dienemann, G. Kokic, H. S. Hillen, C. Höbartner, P. Cramer, Mechanism of molnupiravir-induced SARS-CoV-2 mutagenesis. *Nat. Struct. Mol. Biol.* **28**, 740–746 (2021). doi:10.1038/s41594-

021-00651-0 Medline
12. C. J. Gordon, E. P. Tchesnokov, R. F. Schinazi, M. Götte, Molnupiravir promotes SARS-CoV-2 mutagenesis via the RNA template. *J. Biol. Chem.* **297**, 100770 (2021). doi:10.1016/j.jbc.2021.100770 Medline
13. W. P. Painter, W. Holman, J. A. Bush, F. Almazedi, H. Malik, N. C. J. E. Eraut, M. J. Morin, L. J. Szewczyk, G. R. Painter, Human Safety, Tolerability, and Pharmacokinetics of Molnupiravir, a Novel Broad-Spectrum Oral Antiviral Agent with Activity Against SARS-CoV-2. *Antimicrob. Agents Chemother.* **65**, e02428–e20 (2021). doi:10.1128/AAC.02428-20 Medline
14. G. R. Painter, R. A. Bowen, G. R. Bluemling, J. DeBergh, V. Edpuganti, P. R. Gruddanti, D. B. Guthrie, M. Hager, D. L. Kuiper, M. A. Lockwood, D. G. Mitchell, M. G. Natchus, Z. M. Sticher, A. A. Kolykhalov, The prophylactic and therapeutic activity of a broadly active ribonucleoside analog in a murine model of intranasal venezuelan equine encephalitis virus infection. *Antiviral Res.* **171**, 104597 (2019). doi:10.1016/j.antiviral.2019.104597 Medline
15. M. L. Agostini, A. J. Pruijssers, J. D. Chappell, J. Gribble, X. Lu, E. L. Andres, G. R. Bluemling, M. A. Lockwood, T. P. Sheahan, A. C. Sims, M. G. Natchus, M. Saindane, A. A. Kolykhalov, G. R. Painter, R. S. Baric, M. R. Denison, Small-molecule antiviral β-D-N4-hydroxycytidine inhibits a proofreading-intact coronavirus with a high genetic barrier to resistance. *J. Virol.* **93**, e01348–e19 (2019). doi:10.1128/JVI.01348-19 Medline
16. T. P. Sheahan, A. C. Sims, S. Zhou, R. L. Graham, A. J. Pruijssers, M. L. Agostini, S. R. Leist, A. Schäfer, K. H. Dinnon III, L. J. Stevens, J. D. Chappell, X. Lu, T. M. Hughes, A. S. George, C. S. Hill, S. A. Montgomery, A. J. Brown, G. R. Bluemling, M. G. Natchus, M. Saindane, A. A. Kolykhalov, G. Painter, J. Harcourt, A. Tamin, N. J. Thornburg, R. Swanstrom, M. R. Denison, R. S. Baric, An orally bioavailable broad-spectrum antiviral inhibits SARS-CoV-2 and multiple endemic, epidemic and bat coronavirus. *Sci. Transl. Med.* **12**, eabb5883 (2020). doi:10.1126/scitranslmed.abb5883 Medline
17. A. Wahl, L. E. Gralinski, C. E. Johnson, W. Yao, M. Kovarova, K. H. Dinnon 3rd, H. Liu, V. J. Madden, H. M. Krzystek, C. De, K. K. White, K. Gully, A. Schäfer, T. Zaman, S. R. Leist, P. O. Grant, G. R. Bluemling, A. A. Kolykhalov, M. G. Natchus, F. B. Askin, G. Painter, E. P. Browne, C. D. Jones, R. J. Pickles, R. S. Baric, J. V. Garcia, SARS-CoV-2 infection is effectively treated and prevented by EIDD-2801. *Nature* **591**, 451–457 (2021). doi:10.1038/s41586-021-03312-w Medline
18. A. Jayk Bernal, M. M. Gomes da Silva, D. B. Musungaie, E. Kovalchuk, A. Gonzalez, V. Delos Reyes, A. Martín-Quirós, Y. Caraco, A. Williams-Diaz, M. L. Brown, J. Du, A. Pedley, C. Assaid, J. Strizki, J. A. Grobler, H. H. Shamsuddin, R. Tipping, H. Wan, A. Paschke, J. R. Butterton, M. G. Johnson, C. De Anda; MOVe-OUT Study Group, Molnupiravir for Oral Treatment of Covid-19 in Nonhospitalized Patients. *N. Engl. J. Med.* NEJMoa2116044 (2021). doi:10.1056/NEJMoa2116044 Medline
19. M. D. Folgueira, J. Luczkowiak, F. Lasala, A. Pérez-Rivilla, R. Delgado, Prolonged SARS-CoV-2 cell culture replication in respiratory samples from patients with severe COVID-19. *Clin. Microbiol. Infect.* **27**, 886–891 (2021). doi:10.1016/j.cmi.2021.02.014 Medline
20. A. Singanayagam, M. Patel, A. Charlett, J. Lopez Bernal, V. Saliba, J. Ellis, S. Ladhani, M. Zambon, R. Gopal, Duration of infectiousness and correlation with RT-PCR cycle threshold values in cases of COVID-19, England, January to May 2020. *Euro Surveill.* **25**, 2001483 (2020). doi:10.2807/1560-7917.ES.2020.25.32.2001483 Medline
21. S. Troth, J. Butterton, C. S. DeAnda, P. Escobar, J. Grobler, D. Hazuda, G. Painter, Letter to the Editor in Response to Zhou et al. *J. Infect. Dis.* **224**, 1442–1443 (2021). doi:10.1093/infdis/jiab362 Medline
22. Wisconsin SARS-CoV-2 (hCoV-19) Genomic Dashboard [Internet]. [cited 2021 SEP 28]; Available from: https://dataportal.slh.wisc.edu/sc2dashboard
23. *Regeneron Pharmaceuticals, Inc. Phase 3 Prevention Trial Showed 81% Reduced Risk of Symptomatic SARS-CoV-2 Infections with Subcutaneous Administration of REGEN-COV^{TM} (casirivimab with imdevimab) [Internet].* **Cision PR Newswire** *(2021) [cited 2021 May 12];Available from:* https://www.prnewswire.com/news-releases/phase-3-prevention-trial-showed-81-reduced-risk-of-symptomatic-sars-cov-2-infections-with-subcutaneous-administration-of-regen-cov-casirivimab-with-imdevimab-301266366.html
24. W. Dejnirattisai, D. Zhou, P. Supasa, C. Liu, A. J. Mentzer, H. M. Ginn, Y. Zhao, H. M. E. Duyvesteyn, A. Tuekprakhon, R. Nutalai, B. Wang, C. López-Camacho, J. Slon-Campos, T. S. Walter, D. Skelly, S. A. Costa Clemens, F. G. Naveca, V. Nascimento, F. Nascimento, C. Fernandes da Costa, P. C. Resende, A. Pauvolid-Correa, M. M. Siqueira, C. Dold, R. Levin, T. Dong, A. J. Pollard, J. C. Knight, D. Crook, T. Lambe, E. Clutterbuck, S. Bibi, A. Flaxman, M. Bittaye, S. Belij-Rammerstorfer, S. C. Gilbert, M. W. Carroll, P. Klenerman, E. Barnes, S. J. Dunachie, N. G. Paterson, M. A. Williams, D. R. Hall, R. J. G. Hulswit, T. A. Bowden, E. E. Fry, J. Mongkolsapaya, J. Ren, D. I. Stuart, G. R. Screaton, Antibody evasion by the P.1 strain of SARS-CoV-2. *Cell* **184**, 2939–2954.e9 (2021). doi:10.1016/j.cell.2021.03.055 Medline
25. T. P. Sheahan, A. C. Sims, S. Zhou, R. L. Graham, A. J. Pruijssers, M. L. Agostini, S. R. Leist, A. Schäfer, K. H. Dinnon 3rd, L. J. Stevens, J. D. Chappell, X. Lu, T. M. Hughes, A. S. George, C. S. Hill, S. A. Montgomery, A. J. Brown, G. R. Bluemling, M. G. Natchus, M. Saindane, A. A. Kolykhalov, G. Painter, J. Harcourt, A. Tamin, N. J. Thornburg, R. Swanstrom, M. R. Denison, R. S. Baric, An orally bioavailable broad-spectrum antiviral inhibits SARS-CoV-2 in human airway epithelial cell cultures and multiple coronaviruses in mice. *Sci. Transl. Med.* **12**, eabb5883 (2020). doi:10.1126/scitranslmed.abb5883 Medline
26. R. Abdelnabi, C. S. Foo, S. De Jonghe, P. Maes, B. Weynand, J. Neyts, Molnupiravir Inhibits Replication of the Emerging SARS-CoV-2 Variants of Concern in a Hamster Infection Model. *J. Infect. Dis.* **224**, 749–753 (2021). doi:10.1093/infdis/jiab361 Medline
27. T. Aydillo, A. S. Gonzalez-Reiche, S. Aslam, A. van de Guchte, Z. Khan, A. Obla, J. Dutta, H. van Bakel, J. Aberg, A. García-Sastre, G. Shah, T. Hohl, G. Papanicolaou, M.-A. Perales, K. Sepkowitz, N. E. Babady, M. Kamboj, Shedding of Viable SARS-CoV-2 after Immunosuppressive Therapy for Cancer. *N. Engl. J. Med.* **383**, 2586–2588 (2020). doi:10.1056/NEJMc2031670 Medline
28. A. J. Markmann, N. Giallourou, D. R. Bhowmik, Y. J. Hou, A. Lerner, D. R. Martinez, L. Premkumar, H. Root, D. van Duin, S. Napravnik, S. D. Graham, Q. Guerra, R. Raut, C. J. Petropoulos, T. Wrin, C. Cornaby, J. Schmitz, J. A. Kuruc, S. Weiss, Y. Park, R. Baric, A. M. de Silva, D. M. Margolis, L. A. Bartelt, Sex disparities and neutralizing antibody durability to SARS-CoV-2 infection in convalescent individuals. *MedRxiv Prepr Serv Health Sci* 10.1101/2021.02.01.21250493 (2021).
29. L. Premkumar, B. Segovia-Chumbez, R. Jadi, D. R. Martinez, R. Raut, A. Markmann, C. Cornaby, L. Bartelt, S. Weiss, Y. Park, C. E. Edwards, E. Weimer, E. M. Scherer, N. Rouphael, S. Edupuganti, D. Weiskopf, L. V. Tse, Y. J. Hou, D. Margolis, A. Sette, M. H. Collins, J. Schmitz, R. S. Baric, A. M. de Silva, The receptor binding domain of the viral spike protein is an immunodominant and highly specific target of antibodies in SARS-CoV-2 patients. *Sci. Immunol.* **5**, eabc8413 (2020). doi:10.1126/sciimmunol.abc8413 Medline
30. F. González, N. A. Vielot, M. Sciaudone, C. Toval-Ruíz, L. Premkumar, L. Gutierrez, E. Centeno Cuadra, P. Blandón, A. M. de Silva, R. Rubinstein, N. Bowman, S. Becker-Dreps, F. Bucardo, Seroepidemiology of SARS-CoV-2 infections in an urban Nicaraguan population. *MedRxiv Prepr Serv Health Sci,* 10.1101/2021.02.25.21252447 (2021).

Acknowledgments: The authors acknowledge the participants and the following clinical trial sites for their dedication and contributions to the study: Valley Clinical Trials (Northridge, CA), University of North Carolina (Chapel Hill, NC), Fred Hutchinson Cancer Research Center (Seattle, WA), Care United Research (Forney, TX), Benchmark Research (Colton, CA), FOMAT Medical Research (Oxnard, CA), Indago Research & Health Center (Hiahleah, FL), Wake Forest University (WinstonSalem, NC), Duke University (Durham, NC), and NOLA Research Works (New Orleans, LA). The authors acknowledge Prem Lakshmanane (University of North Carolina at Chapel Hill) for antibody testing and Susanne Henderson (University of North Carolina at Chapel Hill) for study administration support. Additionally, the authors acknowledge the randomization support provided by the National Center for Advancing Translational Sciences (# UL1TR002489) and the UNC Center for AIDS Research (# P30 AI050410). The authors thank Nancy Oglesby for her support in the submission of this manuscript. **Funding:** Ridgeback Biotherapeutics and Merck are jointly developing Molnupiravir. Since licensed by Ridgeback Biotherapeutics, all funds used for the development of Molnupiravir by Ridgeback Biotherapeutics have been provided by Wayne and Wendy Holman and Merck. **Author contributions:** WAF, MC, WPP, WH designed the study. WAF, DAW, JJE, CRW, ERD, MMA ran the clinical trial. RWC conducted RT-PCR of cell culture medium. TPS and RB performed virus isolation studies. WAF, WPP wrote the paper and WAF, WPP, JJE, DAW, TPS, CRW, ERD, RWC reviewed and edited

the paper. **Competing interests:** WAF serves on adjudication committees for Janssen and Syneos and is a consultant to Roche and Merck. WPP has consulted for Drug Innovation Ventures at Emory University and Emory Institute of Drug Development (EIDD). WPP is also an employee of Ridgeback Biotherapeutics. JJE is a consultant to Merck and GlaxoSmithKline and the chair of a data safety monitoring board for Adagio Pharmaceuticals. MSC serves on an advisory board for the Atea Pharmaceutical Company. RB receives funding from the National Institutes of Health on a partnership grant with Gilead Sciences Inc. CRW serves on drug safety monitoring boards for Janssen, Biogen Inc, and Atea Pharmaceutical Company and on advisory boards for Gilead Sciences Inc, Enzychem Lifesciences, Adagio Therapeutics, and Regeneron Pharmaceuticals. DAW serves on advisory boards and consults for Gilead Sciences Inc, ViiV Healthcare, Merck, and Janssen. DAW also receives grant support from Gilead Sciences Inc, ViiV Healthcare, and Merck. WH is the founder and CEO of Ridgeback Capital and a co-owner of Ridgeback Biotherapeutics. WH also owns Merck shares. WH and WPP are, along with others, presently named as coinventors of two pending provisional patent applications entitled "Treatment of Viruses with Antiviral Nucleosides" submitted on behalf of Ridgeback Biopharmaceuticals, Emory University, and Merck Sharp & Dohme Corp. **Data and materials availability:** All data are available in the main text or the supplementary materials. Molnupiravir can be made available to academic researchers through a materials transfer agreement with Ridgeback Biotherapeutics by contacting info@ridgebackbio.com. This work is licensed under a Creative Commons Attribution 4.0 International (CC BY 4.0) license, which permits unrestricted use, distribution, and reproduction in any medium, provided the original work is properly cited. To view a copy of this license, visit https://creativecommons.org/licenses/by/4.0/. This license does not apply to figures/photos/artwork or other content included in the article that is credited to a third party; obtain authorization from the rights holder before using such material.

Submitted 21 August 2021
Accepted 21 December 2021
Published First Release 23 December 2021
10.1126/scitranslmed.abl7430

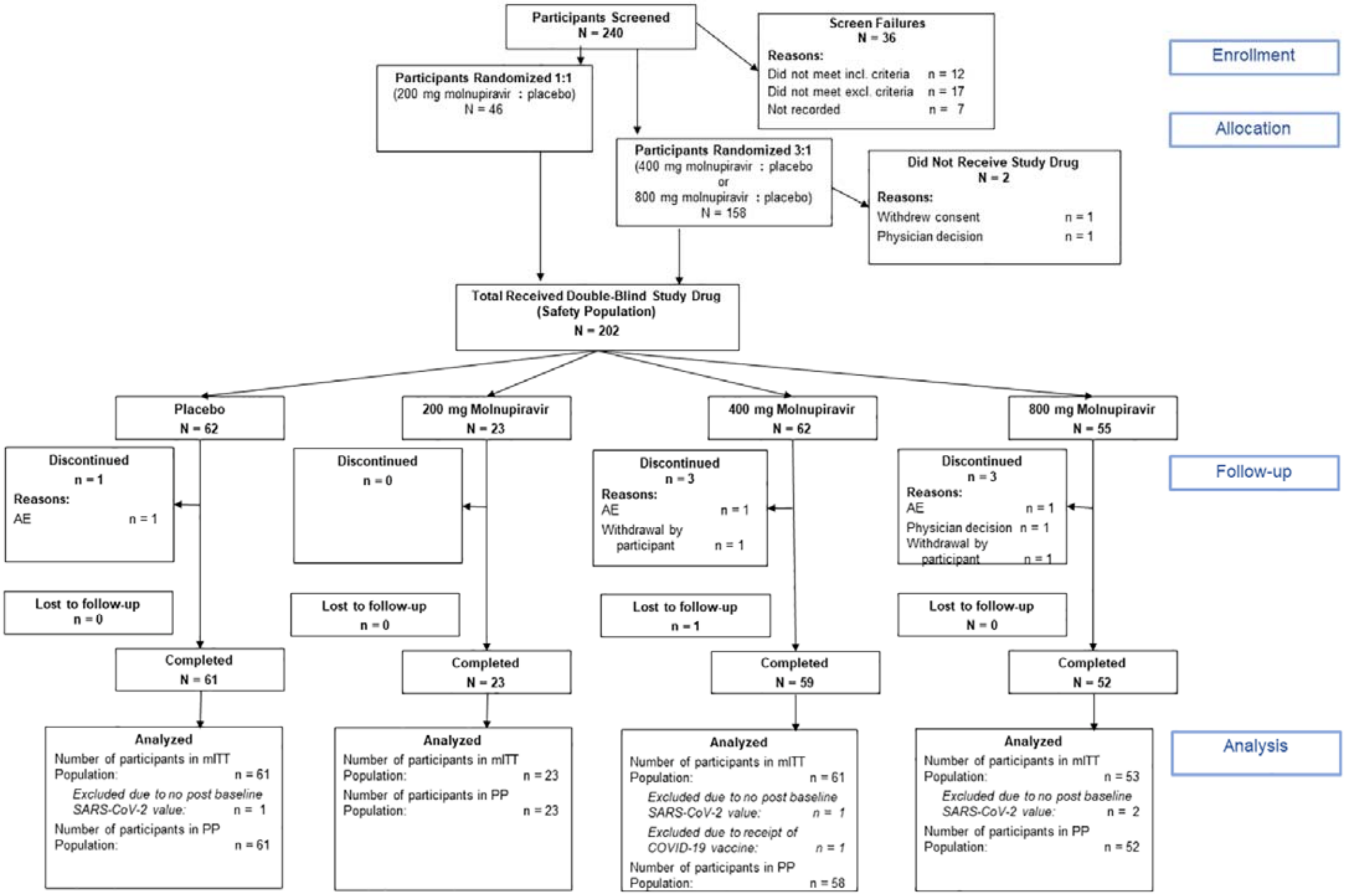

Fig. 1. CONSORT diagram for the Phase 2a trial. Shown is the study flow chart for the Phase 2a randomized, double-blind, placebo-controlled trial of multiple doses of molnupiravir in 202 non-hospitalized adults with recently diagnosed COVID-19 (ClinicalTrials.gov identifier: NCT04405570). Unvaccinated adults aged ≥18 years were eligible if they had a positive test for SARS-CoV-2 infection within 96 hours and had onset of symptoms of COVID-19 within 7 days at the time of treatment initiation (day 1). Antiviral activity, safety, and tolerability of molnupiravir were assessed for 28 days following treatment initiation. The modified intent to treat (mITT) population included all participants who were randomized into the study and had at least one post baseline viral RNA assessment. The per protocol (PP) population included participants in the safety population who had no important protocol deviations leading to exclusion from the per protocol population and had completed the day 28 followup visit. The safety population included all participants who were randomized and took at least one dose of the study drug. Participants were analyzed according to the treatment they actually received. AE, adverse events.

Fig. 2. SARS-CoV-2 Infectivity and Virology. **(A)** Shown is a Kaplan-Meier plot of time to clearance of SARS-CoV-2 RNA by treatment: 200 mg molnupiravir (red), 400 mg molnupiravir (blue), 800 mg molnupiravir (cyan), or placebo (black). **(B)** Shown is the percentage of participants who were positive (red) for SARS-CoV-2 infectious virus at day 1 (baseline), day 3 and day 5 of treatment. Participants who were negative for SARS-CoV-2 infectious virus are in blue. Comparison was based on pooled placebo data. **(C)** Shown is the least squares mean change from baseline in SARS-CoV-2 RNA (log_{10} copies/mL) for the modified intent to treat population at study days 3,5,7, and 14: 200 mg molnupiravir (red), 400 mg molnupiravir (blue), 800 mg molnupiravir (cyan), or placebo (black).

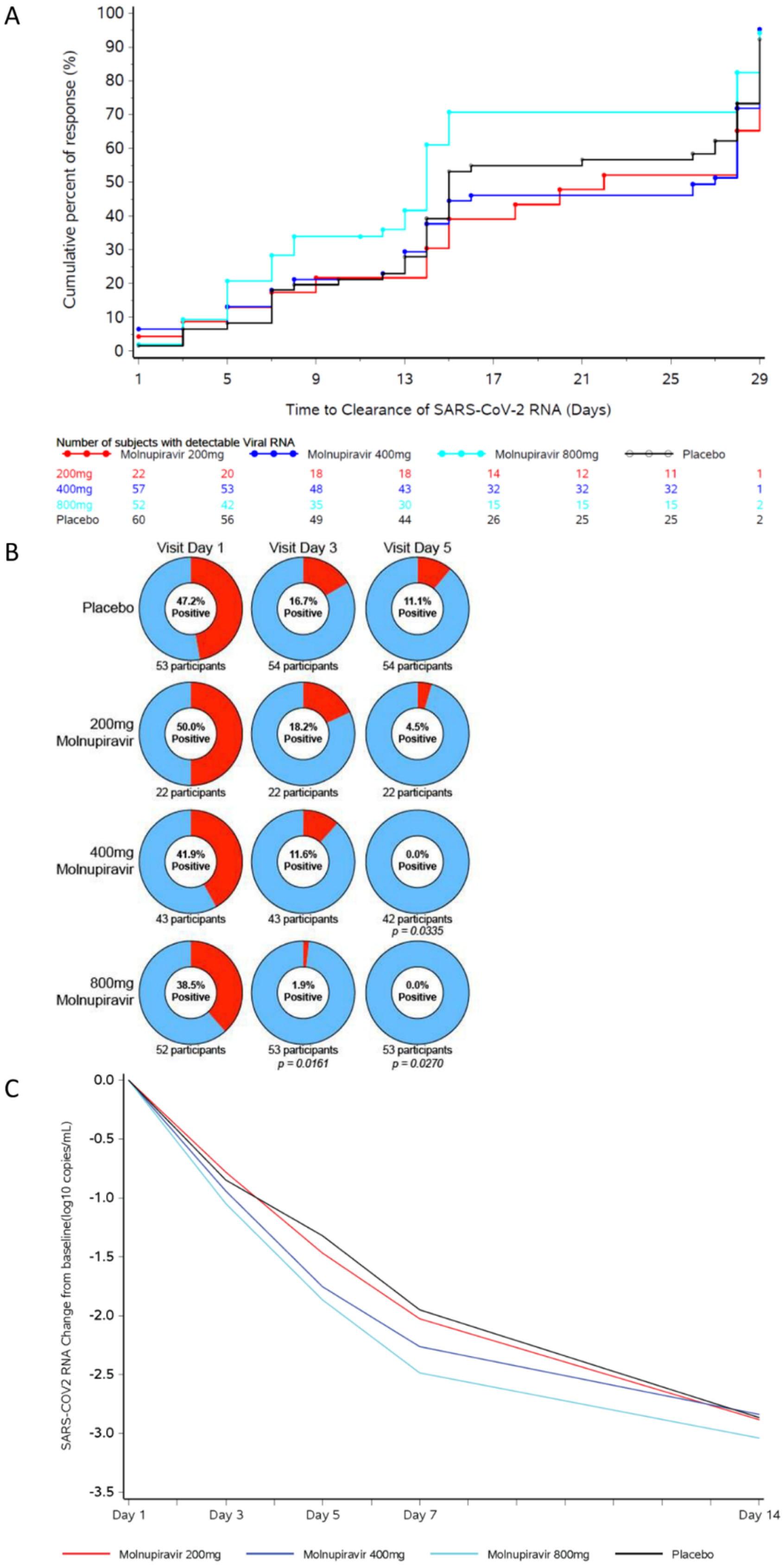

Table 1. Patient Demographics and Baseline Characteristics.

	200 mg Molnupiravir N = 23	400 mg Molnupiravir N = 62	800 mg Molnupiravir N = 55	Placebo N = 62
Age, median (range), years	32.0 (19 - 65)	42.5 (19 - 82)	42.0 (18 - 68)	39.0 (19 - 71)
Age ≥65 years, n (%)	1 (4.3)	3 (4.8)	4 (7.3)	3 (4.8)
Sex, female, n (%)	11 (47.8)	32 (51.6)	27 (49.1)	34 (54.8)
Race, n (%)				
Asian	1 (4.3)	2 (3.2)	1 (1.8)	2 (3.2)
Black or African American	3 (13.0)	3 (4.8)	3 (5.5)	2 (3.2)
White	17 (73.9)	56 (90.3)	49 (89.1)	54 (87.1)
Other	2 (8.7)	0	2 (3.6)	1 (1.6)
Multiple	0	1 (1.6)	0	3 (4.8)
Ethnicity, Hispanic or Latino, n (%)	7 (30.4)	23 (37.1)	33 (60.0)	23 (37.1)
BMI, median, kg/m^2	25.50	26.70	27.00	27.05
BMI ≥30 kg/m^2, n (%)	7 (30.4)	18 (29.0)	15 (27.3)	16 (25.8)
Baseline viral load, mean (SD), log_{10} copies/mL	6.69 (1.888)	6.38 (1.837)	5.80 (1.823)	6.11 (1.794)
Baseline viral load, median (range), log_{10} copies/mL	7.25 (3.0 – 9.5)	6.72 (3.0 – 9.9)	6.12 (3.0 – 9.4)	6.40 (3.0 – 9.3)
Days from symptom onset, median (range), n	4.00 (1.8 – 7.0)	4.85 (2.5 – 7.1)	4.60 (1.4 – 7.1)	4.55 (1.8 – 7.5)
At least 1 risk factor for severe disease, n (%)	15 (65.2)	37 (59.7)	33 (60.0)	37 (59.7)
SARS-CoV-2 Spike protein antibody positive** on day 1, n/N* (%)	3/20 (15.0)	15/50 (30.0)	18/51 (35.3)	10/55 (18.2)

BMI, body mass index; n, number of participants with response; N, number of participants in dose group; OD, optical density; SD, standard deviation.
*N, number of participants with an antibody status.
** SARS-CoV-2 Spike protein antibody positive includes testing positive for any of the following: Total Ig, IgA, IgM, or IgG. A positive result was defined as an OD reading greater than 0.376 for total Ig, 0.3 for IgA, 0.31 for IgM, and 0.376 for IgG.

Table 2. SARS-CoV-2 Infectivity and Virology.

**Percentage of Participants Positive for Infectious SARS-CoV-2 Virus**				
	**200 mg Molnupiravir**	**400 mg Molnupiravir**	**800 mg Molnupiravir**	**Placebo**
Day 1, n/N (%)	11/22 (50.0)	18/43 (41.9)	20/52 (38.5)	25/53 (47.2)
Day 3, n/N (%)	4/22 (18.2)	5/43 (11.6)	1/53 (1.9)	9/54 (16.7)
*Fishers exact p-value**	>0.99	0.57	0.016	
Dose response p-value				0.010
Day 5, n/N (%)	1/22 (4.5)	0/42 (0.0)	0/53 (0.0)	6/54 (11.1)
*Fishers exact p-value**	0.67	0.034	0.027	
Dose response p-value				0.003
**Time to SARS-CoV-2 Viral RNA Negativity**				
	**200 mg Molnupiravir**	**400 mg Molnupiravir**	**800 mg Molnupiravir**	**Placebo**
Participants with Response, n/N (%)	21/23 (91.3)	48/61 (78.7)	49/53 (92.5)	49/61 (80.3)
Median time to response (95% CI), days	22.0 (15.0, 28.0)	27.0 (15.0, 28.0)	14.0 (13.0, 14.0)	15.0 (15.0, 27.0)
*Log-rank p-value**	0.56	0.73	0.013	
**Change from Baseline in SARS-CoV-2 Viral Load (log_{10} copies/mL)**				
	**200 mg Molnupiravir**	**400 mg Molnupiravir**	**800 mg Molnupiravir**	**Placebo**
Day 3, n/N	23/23	58/61	51/53	56/61
Least squares mean (SE)	-0.783 (0.189)	-0.941 (0.118)	-1.050 (0.115)	-0.847 (0.127)
Difference in least squares mean	0.064	-0.094	-0.203	
95% CI	-0.397, 0.525	-0.438, 0.250	-0.543, 0.137	
p-value	0.78	0.59	0.24	
Day 5, n/N	23/23	56/61	52/53	57/61
Least squares mean (SE)	-1.471 (0.212)	-1.754 (0.128)	-1.867 (0.126)	-1.320 (0.150)
Difference in least squares mean	-0.150	-0.434	-0.547	
95% CI	-0.674, 0.374	-0.825, -0.043	-0.935, -0.159	
p-value	0.57	0.030	0.006	
Day 7, n/N	23/23	51/61	49/53	56/61
Least squares mean (SE)	-2.028 (0.202)	-2.263 (0.119)	-2.485 (0.107)	-1.952 (0.157)
Difference in least squares mean	-0.076	-0.311	-0.534	
95% CI	-0.591, 0.438	-0.702, 0.079	-0.910, -0.157	
p-value	0.77	0.12	0.006	
Day 14, n/N	23/23	53/61	48/53	54/61
Least squares mean (SE)	-2.884 (0.091)	-2.840 (0.065)	-3.040 (0.040)	-2.865 (0.106)
Difference in least squares mean	-0.019	0.026	-0.175	
95% CI	-0.298, 0.260	-0.220, 0.272	-0.400, 0.050	
p-value	0.89	0.84	0.13	

CI, confidence interval; n, number of observations; N, number of participants; SE, standard error.
* Comparison based on pooled placebo data

Table 3. Adverse Events.

Number (%) of participants experiencing an adverse event	Molnupiravir 200 mg N = 23	Molnupiravir 400 mg N = 62	Molnupiravir 800 mg N = 55	Placebo N = 62
Any adverse event	11 (47.8)	20 (32.3)	11 (20.0)	18 (29.0)
Adverse events reported by >5% subjects in any group				
Dizziness	2 (8.7)	1 (1.6)	0	0
Insomnia	2 (8.7)	1 (1.6)	1 (1.8)	4 (6.5)
Any adverse event grade 3 or higher	1 (4.3)	2 (3.2)	4 (7.3)	5 (8.1)
Any adverse event leading to discontinuation of study drug	0	1 (1.6)	1 (1.8)	1 (1.6)
Any adverse event related to study drug	4 (17.4)	13 (21.0)	1 (1.8)	8 (12.9)
Any serious adverse event	0	2 (3.2)	1 (1.8)	1 (1.6)
Any adverse event leading to death	0	0	0	1 (1.6)*

N, number of participants in dose group.
***** The subject had an adverse event of hypoxia that led to death. This occurred 31 days after discontinuation from the study following completion of study assessments and was not recorded in the study database but was recorded in the safety database.
