# Supplementary material for: A phase 2a clinical trial of molnupiravir in patients with COVID-19 shows accelerated SARS-CoV-2 RNA clearance and elimination of infectious virus
Source: Sci Transl Med. 2022 Jan 19;14(628):eabl7430. doi: 10.1126/scitranslmed.abl7430 (PMC10763622; doi:10.1126/scitranslmed.abl7430)
Supplement: Supplementary file 2 — Tables S1 to S11 [file scitranslmed.abl7430_sm.pdf]

Science Translational Medicine
AAAS

Supplementary Materials for

A phase 2a clinical trial of molnupiravir in patients with COVID-19 shows accelerated SARS-CoV-2 RNA clearance and elimination of infectious virus

William A. Fischer II *et al.*

Corresponding author: William A. Fischer II, william_fischer@med.unc.edu;
Wendy P. Painter, painter@ridgebackbio.com

Sci. Transl. Med. **14**, eabl7430 (2022)
DOI: 10.1126/scitranslmed.abl7430

The PDF file includes:

Tables S1 to S11

Other Supplementary Material for this manuscript includes the following:

Reproducibility Checklist

Table S1. Time to SARS-CoV-2 Viral RNA Negativity in Participants Who Were Negative for Antibodies at Baseline

	200 mg Molnupiravir	400 mg Molnupiravir	800 mg Molnupiravir	Placebo
Participants with Response, n/N (%)	16/17 (94.1)	26/34 (76.5)	32/32 (100)	34/44 (77.3)
Median time to response (95% CI), days	22.0 (15.0, 29.0)	26.0 (15.0, 28.0)	14.0 (13.0, 14.0)	27.0 (15.0, 28.0)
Log-rank p-value	0.87	0.48	0.001	

Abbreviations: CI = confidence interval; n = number of observations; N = number of participants; SARS-CoV-2 = severe acute respiratory syndrome coronavirus 2.

Table S2. Summary of Treatment Emergent Adverse Events by System Organ Class and Preferred Term (Safety Population)

	Molnupiravir 200 mg	Molnupiravir 400 mg	Molnupiravir 800 mg	Placebo
**System Organ Class**	**(N=23)**	**(N=62)**	**(N=55)**	**(N=62)**
**Preferred Term**	**n (%) E**	**n (%) E**	**n (%) E**	**n (%) E**
Subjects with at Least 1 Adverse Event	11 (47.8) 16	20 (32.3) 42	11 (20.0) 21	18 (29.0) 31
Nervous system disorders	4 (17.4) 4	6 (9.7) 10	2 (3.6) 2	3 (4.8) 4
Headache	1 (4.3) 1	3 (4.8) 4	2 (3.6) 2	3 (4.8) 3
Dizziness	2 (8.7) 2	1 (1.6) 3	0	0
Paraesthesia	0	2 (3.2) 2	0	0
Cerebrovascular accident	0	1 (1.6) 1	0	0
Syncope	1 (4.3) 1	0	0	0
Migraine	0	0	0	1 (1.6) 1
Investigations	2 (8.7) 3	5 (8.1) 8	3 (5.5) 6	4 (6.5) 6
Alanine aminotransferase increased	0	2 (3.2) 2	2 (3.6) 2	2 (3.2) 2
Aspartate aminotransferase increased	0	1 (1.6) 1	2 (3.6) 2	1 (1.6) 1
Blood creatinine increased	0	3 (4.8) 3	0	0
Blood alkaline phosphatase increased	0	0	1 (1.8) 1	0
Blood glucose decreased	1 (4.3) 1	0	0	1 (1.6) 1
Blood glucose increased	0	0	1 (1.8) 1	0
Creatinine renal clearance decreased	1 (4.3) 2	0	0	1 (1.6) 1
Haemoglobin decreased	0	1 (1.6) 1	0	0
Oxygen saturation decreased	0	1 (1.6) 1	0	0
Blood pressure increased	0	0	0	1 (1.6) 1
Gastrointestinal disorders	1 (4.3) 1	7 (11.3) 8	0	5 (8.1) 6
Nausea	1 (4.3) 1	2 (3.2) 2	0	1 (1.6) 1
Abdominal pain	0	1 (1.6) 1	0	2 (3.2) 2
Abdominal pain upper	0	1 (1.6) 1	0	1 (1.6) 1
Diarrhoea	0	1 (1.6) 1	0	1 (1.6) 1
Gastrooesophageal reflux disease	0	1 (1.6) 1	0	1 (1.6) 1
Hyperaesthesia teeth	0	1 (1.6) 1	0	0
Oral mucosal exfoliation	0	1 (1.6) 1	0	0
Respiratory, thoracic and mediastinal disorders	1 (4.3) 1	2 (3.2) 3	2 (3.6) 2	1 (1.6) 1
Cough	0	2 (3.2) 2	1 (1.8) 1	0
Acute respiratory failure	0	0	1 (1.8) 1	0
Asthma	1 (4.3) 1	0	0	0
Dyspnoea	0	1 (1.6) 1	0	0
Hypoxia	0	0	0	1 (1.6) 1
Psychiatric disorders	2 (8.7) 2	1 (1.6) 1	1 (1.8) 2	4 (6.5) 4
Insomnia	2 (8.7) 2	1 (1.6) 1	1 (1.8) 2	4 (6.5) 4

General disorders and administration site conditions	0	2 (3.2) 4	1 (1.8) 2	3 (4.8) 3
Chest discomfort	0	1 (1.6) 2	0	1 (1.6) 1
Chills	0	0	1 (1.8) 1	0
Pyrexia	0	0	1 (1.8) 1	0
Vessel puncture site bruise	0	1 (1.6) 2	0	0
Non-cardiac chest pain	0	0	0	1 (1.6) 1
Peripheral swelling	0	0	0	1 (1.6) 1
Infections and infestations	0	2 (3.2) 2	1 (1.8) 1	1 (1.6) 1
Pneumonia	0	1 (1.6) 1	1 (1.8) 1	0
Oral herpes	0	1 (1.6) 1	0	0
Genital herpes	0	0	0	1 (1.6) 1
Musculoskeletal and connective tissue disorders	1 (4.3) 1	1 (1.6) 1	1 (1.8) 1	1 (1.6) 2
Arthralgia	0	1 (1.6) 1	0	0
Back pain	1 (4.3) 1	0	0	0
Myalgia	0	0	1 (1.8) 1	1 (1.6) 1
Musculoskeletal chest pain	0	0	0	1 (1.6) 1
Skin and subcutaneous tissue disorders	0	2 (3.2) 3	1 (1.8) 1	2 (3.2) 2
Rash	0	1 (1.6) 1	1 (1.8) 1	0
Pruritus	0	1 (1.6) 1	0	1 (1.6) 1
Skin burning sensation	0	1 (1.6) 1	0	0
Hyperhidrosis	0	0	0	1 (1.6) 1
Cardiac disorders	0	1 (1.6) 1	1 (1.8) 1	0
Cardiac flutter	0	1 (1.6) 1	0	0
Supraventricular tachycardia	0	0	1 (1.8) 1	0
Ear and labyrinth disorders	2 (8.7) 2	0	0	1 (1.6) 1
Ear pain	1 (4.3) 1	0	0	0
Vertigo	1 (4.3) 1	0	0	0
Ear discomfort	0	0	0	1 (1.6) 1
Injury, poisoning and procedural complications	1 (4.3) 2	0	1 (1.8) 1	1 (1.6) 1
Accidental overdose	0	0	1 (1.8) 1	0
Arthropod bite	1 (4.3) 1	0	0	0
Muscle strain	1 (4.3) 1	0	0	0
Alcohol poisoning	0	0	0	1 (1.6) 1
Blood and lymphatic system disorders	0	0	1 (1.8) 1	0
Anaemia	0	0	1 (1.8) 1	0
Eye disorders	0	0	1 (1.8) 1	0
Blepharitis	0	0	1 (1.8) 1	0
Metabolism and nutrition disorders	0	1 (1.6) 1	0	0
Hyponatraemia	0	1 (1.6) 1	0	0

n = number of participants; E = number of events.

Table S3. Summary of SARS-CoV-2 Infectivity for Participants Who Had Infectious Virus Isolation at Baseline

	**200 mg Molnupiravir** N = 11	**400 mg Molnupiravir** N = 18	**800 mg Molnupiravir** N = 20	**Placebo** N = 25
Day 3, n/N (%)	4/11 (36.4)	5/18 (27.8)	1/20 (5.0)	7/25 (28.0)
Fishers exact p-value	0.70	>0.99	0.06	
Dose response p-value				0.06
Day 5, n/N (%)	1/11 (9.1)	0/18 (0.0)	0/20 (0.0)	6/25 (24.0)
Fishers exact p-value	0.40	0.03	0.03	
Dose response p-value				0.004

Abbreviations: n = number of observations; N = number of participants; SARS-CoV-2 = severe acute respiratory syndrome coronavirus 2.

Table S4. Summary of SARS-CoV-2 Infectivity for Participants Who Had Infective Virus Isolation at Baseline and Were Negative for Antibodies

	200 mg Molnupiravir N = 9	400 mg Molnupiravir N = 17	800 mg Molnupiravir N = 20	Placebo N = 24
Day 3, n/N (%)	2/9 (22.2)	5/17 (29.4)	1/20 (5.0)	7/24 (29.2)
Fishers exact p-value	>0.99	>0.99	0.05	
Dose response p-value				0.07
Day 5, n/N (%)	0/9 (0.0)	0/17 (0.0)	0/20 (0.0)	6/24 (25.0)
Fishers exact p-value	0.16	0.03	0.02	
Dose response p-value				0.003

Abbreviations: n = number of observations; N = number of participants; SARS-CoV-2 = severe acute respiratory syndrome coronavirus 2.

Table S5. Summary of SARS-CoV-2 Infectious Virus Isolation: 800 mg Molnupiravir Compared with Concurrent Placebo Recipients

Number of Positive Subjects	800 mg Molnupiravir	Placebo
Day 1, n/N (%)	20/52 (38.5%)	8/17 (47.1)
p-value	0.58	
Day 3, n/N (%)	1/53 (1.9)	2/18 (11.1)
p-value	0.16	
Day 5, n/N (%)	0/53 (0.0)	3/18 (16.7)
p-value	0.014	
Day 7, n/N (%)	0/52 (0.0)	1/18 (5.6)
p-value	0.26	

Abbreviations: n = number of observations; N = number of participants.

Table S6. Change from Baseline in SARS-CoV-2 Viral Load (log_{10} copies/mL) in Participants Who Were Negative for Antibodies at Baseline

	**200 mg Molnupiravir**	**400 mg Molnupiravir**	**800 mg Molnupiravir**	**Placebo**
Day 3, n/N	17/17	34/34	31/32	44/44
Least squares mean (SE)	-1.141 (0.183)	-1.196 (0.132)	-1.157 (0.129)	-0.877 (0.150)
Difference in least squares mean	-0.263	-0.319	-0.280	
95% CI	-0.734, 0.207	-0.714, 0.076	-0.670, 0.111	
p-value	0.27	0.11	0.16	
Day 5, n/N	17/17	34/34	31/32	44/44
Least squares mean (SE)	-1.874 (0.183)	-2.101 (0.132)	-2.191 (0.129)	-1.578 (0.150)
Difference in least squares mean	-0.296	-0.523	-0.613	
95% CI	-0.767, 0.174	-0.917, -0.128	-1.004, -0.223	
p-value	0.22	0.010	0.002	
Day 7, n/N	17/17	31/34	31/32	43/44
Least squares mean (SE)	-2.447 (0.183)	-2.582 (0.137)	-2.967 (0.129)	-2.284 (0.152)
Difference in least squares mean	-0.163	-0.298	-0.683	
95% CI	-0.635, 0.308	-0.702, 0.105	-1.075, -0.290	
p-value	0.50	0.15	<0.001	
Day 14, n/N	17/17	32/34	31/32	41/44
Least squares mean (SE)	-3.579 (0.183)	-3.412 (0.136)	-3.601 (0.129)	-3.343 (0.155)
Difference in least squares mean	-0.236	-0.069	-0.257	
95% CI	-0.711, 0.239	-0.475, 0.337	-0.654, 0.140	
p-value	0.33	0.74	0.20	

Abbreviations: CI = confidence interval; n = number of observations; N = number of participants; SARS-CoV-2 = severe acute respiratory syndrome coronavirus 2; SE = standard error.

Table S7. Change from Baseline in SARS-CoV-2 Viral Load (log_{10} copies/mL)

	**800 mg Molnupiravir** N = 53	**Placebo** N = 18
Day 3, n/N	51/53	16/18
Least squares mean (SE)	-0.955 (0.094)	-0.638 (0.166)
Difference in least squares mean	-0.316	
95% CI	-0.696, 0.063	
p-value	0.101	
Day 5, n/N	52/53	17/18
Least squares mean (SE)	-1.673 (0.093)	-1.297 (0.160)
Difference in least squares mean	-0.376	
95% CI	-0.744, -0.008	
p-value	0.045	

Abbreviations: CI = confidence interval; n = number of observations; N = number of participants; SARS-CoV-2 = severe acute respiratory syndrome coronavirus 2; SE = standard error.

Table S8. Summary of Time to Patient Reported General Physical Health At least Very Good (Safety Population)

	Pooled Molnupiravir Groups (N=140)	Placebo (N=62)
Number (%) Subjects with Response:	90 (64.3)	41 (66.1)
Time to Response (days)		
25 % (95% CI)	8.0 (6.0, 9.0)	6.0 (5.0, 9.0)
Median (95% CI)	14.0 (13.0, 21.0)	15.0 (12.0, 18.0)
75 % (95% CI)	NE	NE
Log Rank p-value	0.5856	

Abbreviations: CI = confidence interval; N = number of participants; NE = not estimated

Table S9. Summary of COVID-19-Related Medical Visits (Safety Population)

	Molnupiravir 200 mg (N=23)	Molnupiravir 400 mg (N=62)	Molnupiravir 800 mg (N=55)	Placebo (N=62)
	**n (%)**	**n (%)**	**n (%)**	**n (%)**
Participants with a COVID-19-related medical visit				
Yes	3 (13.0)	4 (6.5)	5 (9.1)	5 (8.1)
No	20 (87.0)	58 (93.5)	50 (90.9)	57 (91.9)
p-value	0.6768	>.9999	>.9999	

Abbreviations: COVID-19 = coronavirus disease 2019; n = number of observations; N = number of participants

Table S10. Missing Data for SARS-CoV-2 Infectious Virus

Visit Before	Visit After	Imputation of Infectivity Result
Negative	Negative	Negative
Positive	Positive	Positive
Negative or missing	Positive	Positive*
Missing	Negative	Missing
Any value	Missing	Missing

* For participants with missing baseline assessments and reported the first post baseline value as positive, the baseline was imputed as positive.

Table S11. Missing Data for Time to SARS-CoV-2 Viral RNA Negativity

Visit Before	Visit After	Imputation of Undetectable SARS-CoV-2 RNA
BLQ/BLD	BLD or BLQ	Negative
>BLQ	BLD or BLQ	Positive
BLD/BLQ or missing	>BLQ	Positive*
Missing	BLD or BLQ	Missing
Any value	Missing	Missing

Abbreviations: BLD = below limit of detection; BLQ = below limit of quantification; SARS-CoV-2 = severe acute respiratory syndrome coronavirus 2.

* For participants with missing baseline assessments who reported the first post baseline value as >BLQ, the baseline was imputed as >BLQ (positive).
